# Identification of nonhistone substrates of the lysine methyltransferase PRDM9

**DOI:** 10.1016/j.jbc.2023.104651

**Published:** 2023-03-25

**Authors:** Jocelyne N. Hanquier, Kenidi Sanders, Christine A. Berryhill, Firoj K. Sahoo, Andy Hudmon, Jonah Z. Vilseck, Evan M. Cornett

**Affiliations:** 1 Department of Biochemistry and Molecular Biology, Indiana University School of Medicine, Indianapolis, Indiana, USA; 2 Stark Neuroscience Research Institute, Indiana University School of Medicine, Indianapolis, Indiana, USA; 3 Melvin and Bren Simon Comprehensive Cancer Center, Indiana University School of Medicine, Indianapolis, Indiana, USA; 4 Department of Medicinal Chemistry and Molecular Pharmacology, College of Pharmacy, Purdue University, West Lafayette, Indiana, USA; 5 Center for Computational Biology and Bioinformatics, Indiana University School of Medicine, Indianapolis, Indiana, USA

**Keywords:** histone modifications, post-translational modifications, lysine methylation, methyltransferases, substrate selectivity, peptide libraries

## Abstract

Lysine methylation is a dynamic, posttranslational mark that regulates the function of histone and nonhistone proteins. Many of the enzymes that mediate lysine methylation, known as lysine methyltransferases (KMTs), were originally identified to modify histone proteins but have also been discovered to methylate nonhistone proteins. In this work, we investigate the substrate selectivity of the KMT PRDM9 to identify both potential histone and nonhistone substrates. Though normally expressed in germ cells, PRDM9 is significantly upregulated across many cancer types. The methyltransferase activity of PRDM9 is essential for double-strand break formation during meiotic recombination. PRDM9 has been reported to methylate histone H3 at lysine residues 4 and 36; however, PRDM9 KMT activity had not previously been evaluated on nonhistone proteins. Using lysine-oriented peptide libraries to screen potential substrates of PRDM9, we determined that PRDM9 preferentially methylates peptide sequences not found in any histone protein. We confirmed PRDM9 selectivity through *in vitro* KMT reactions using peptides with substitutions at critical positions. A multisite λ-dynamics computational analysis provided a structural rationale for the observed PRDM9 selectivity. The substrate selectivity profile was then used to identify putative nonhistone substrates, which were tested by peptide spot array, and a subset was further validated at the protein level by *in vitro* KMT assays on recombinant proteins. Finally, one of the nonhistone substrates, CTNNBL1, was found to be methylated by PRDM9 in cells.

Dynamic lysine methylation, added by lysine methyltransferases (KMTs) and removed by lysine demethylases, regulates the function of both histone and nonhistone proteins (1, 2). Many KMTs have been studied in the context of histone lysine methylation, which is well understood to regulate chromatin-templated processes. However, many of the KMTs initially identified to modify histone proteins have since been discovered to also methylate nonhistone protein substrates (1, 2). Furthermore, several studies have revealed that a large portion of the human proteome is modified with lysine methylation (3, 4, 5). To connect KMTs with their substrates, we previously developed a functional proteomics approach to map KMT substrate selectivity using lysine-oriented peptide libraries (K-OPLs) (6). In this study, we use this approach to characterize the substrate selectivity of the KMT domain of PRDM9.

PRDM9 is a member of the PR domain–containing family of KMTs that contain a PR domain coupled with an array of C2H2 zinc fingers (7, 8, 9). The PR domain is closely related to the SET domain (10), conferring methyltransferase activity, while the zinc fingers allow PRDM proteins to bind to DNA in a sequence-specific manner (11). PRDM9 was first identified as a sterility factor in mice and humans (12, 13). The DNA-binding activity of PRDM9 coincides with hotspots of meiotic recombination (14), and PRDM9 methyltransferase activity is essential for double-strand break formation at PRDM9-designated recombination sites (15). Initial characterization of PRDM9 found that it is typically expressed in female ovaries during development but was only detected in adult testis (13). More recently, an analysis of human patient cancer samples revealed a significant upregulation of PRDM9 across many cancer types (16). However, understanding the role of PRMD9 in these cancer types has been limited by incomplete knowledge of PRDM9 substrates.

The methyltransferase activity of PRDM9 toward histone proteins has been extensively characterized. PRDM9 was first reported to trimethylate H3K4 (H3K4me3). However, this initial characterization relied upon *in vitro* methyltransferase assays using mouse Prdm9 (herein referred to as mPrdm9; human referred to as PRDM9) and histone substrates isolated from calf thymus and transient overexpression of mPrdm9 in COS-7 cells, with site-specific antibodies used for a limited number of modifications on histone H3 (13). More in-depth studies, including *in vitro* KMT assays followed by mass spectrometry, revealed that mPrdm9 is also capable of H3K4 mono-methylation (H3K4me1) and di-methylation (H3K4me2) (17, 18, 19). Experiments using histone peptide arrays also implicated H3K36 as a potential substrate, which was later validated by Eram *et al.* (18) using *in vitro* reactions on both peptides and nucleosomes. Additionally, overexpression of PRDM9 in HEK293T cells increased the global levels of both H3K4me3 and H3K36me3 *in vivo* (18). Another study has suggested that PRDM9 may have substrates on other histone proteins and showed that mPrdm9 mediated the methylation of all four core histone units—H3, H4, H2A, and H2b—alone and within the histone octamer (17). To our knowledge, PRDM9 activity has not been evaluated on nonhistone proteins before. In this work, we investigate the substrate selectivity of PRDM9, and one of our key findings is that PRDM9 prefers to methylate peptide sequences not found in any histone protein.

## Results and discussion

### PRDM9 prefers sequence motifs not found in any histone protein

**Figure 1:**
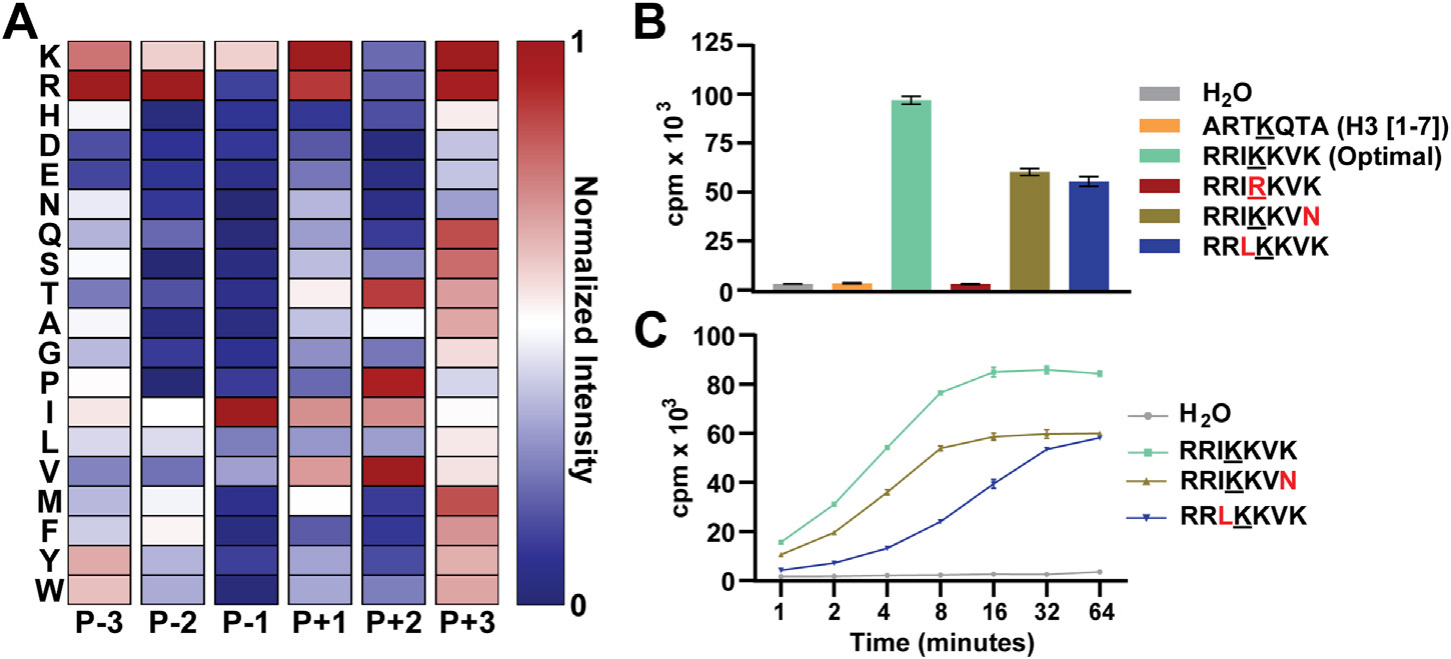
**Substrate selectivity of PRDM9.***A*, K-OPL substrate selectivity profile for PRDM9. Averaged results from three independent K-OPL surface proximity assay screens for PRDM9 depicted as a position-normalized heat map (Fig. S1 for globally normalized heat map and raw K-OPL data); the exception is duplicate measurements for P+2 M. The color code is proportional to the creation of enzyme product, where *red* (1) is most active and *blue* (0) is least active. Rows show the identity of each fixed residue, and columns show the position within the sequence. *B*, validation of PRDM9 substrate selectivity using peptides with substitutions to critical residues. KMT reactions consisted of 0.8 μg PRDM9, 0.5 μg substrate, and 2 μCi SAM and were incubated for 1 h at room temperature. Graph displays mean (n = 3) ± SD. *C*, initial rate measurements with the optimal PRDM9 substrate (RRIKKVK, *bright green*) and peptides containing substitutions at P-1 (I to L, *blue*) and P+3 (K to N, *moss green*) positions. KMT reactions consisted of 0.8 μg PRDM9, 0.5 μg substrate, and 2 μCi SAM. Graph displays mean (n = 3) ± SD; error bars are masked by the symbol weight for some data points. Cpm, counts per minute; KMT, lysine methyltransferases; K-OPL, lysine-oriented peptide library; SAM, S-adenosyl methionine.

To determine the preferred sequence determinants of PRDM9, we screened a K-OPL to query ∼64 million unique peptide sequences split into 114 sets. Each set contained a fixed central lysine residue (P0) with an additional fixed amino acid within three positions of the central lysine. Transfer of tritium-labeled methyl groups from S-adenosyl methionine (SAM) to the biotin-labeled K-OPL peptides was detected using a sensitive surface proximity assay (6). Three independent reactions for each of the 114 separate sets were averaged and normalized to the highest signal across all sets for each amino acid fixed at a particular position relative to the central lysine residue (Fig. 1*A*). The selectivity analysis reveals that PRDM9 prefers isoleucine (Ile; I) in the P-1 position. To confirm the signal of PRDM9 on the K-OPL peptides, we selected several K-OPL sets with high, medium, or low signal (see Experimental procedures) at three different positions and performed additional methyltransferase assays confirming the preference for each amino acid at these positions (Fig. S1*C*). To check the potential impact of neighboring posttranslational modifications on PRDM9 activity, we also performed analysis of additional K-OPL sets that contained phosphorylated serine (Ser; S), tyrosine (Tyr; Y), or threonine (Thr; T). PRDM9 showed a preference for Ser in the P-3 position and Thr in the P+2 position. Phosphorylation of these residues reduced activity to near background levels (Fig. S1*D*), highlighting the potential for posttranslational modification cross talk (1).

To validate the PRDM9 substrate selectivity profile, a series of peptides were synthesized based on the optimal substrate (RRIKKVK). PRDM9 methylated the optimal substrate nearly 100-fold more efficiently than a peptide containing H3K4 (Fig. 1*B*). As predicted, the substitution of the P-1 Ile with leucine (Leu; L) resulted in a significant reduction in PRDM9 methyltransferase activity (Fig. 1, *B* and *C*). Importantly, there was no signal when using a peptide with the central lysine (Lys; K) substituted with arginine (Arg; R) (Fig. 1*B*). The position normalized and globally normalized heatmaps of PRDM9 substrate selectivity (Figs. 1*A* and S1*B*) suggest PRDM9 has a stronger preference for the P-1 position than P+3. To evaluate this, we also tested a peptide with lysine substituted with asparagine (Asn; N) in the P+3 position, which resulted in reduced activity but had less impact than the substitution made in the P-1 position (Fig. 1, *B* and *C*).

**Figure 2:**
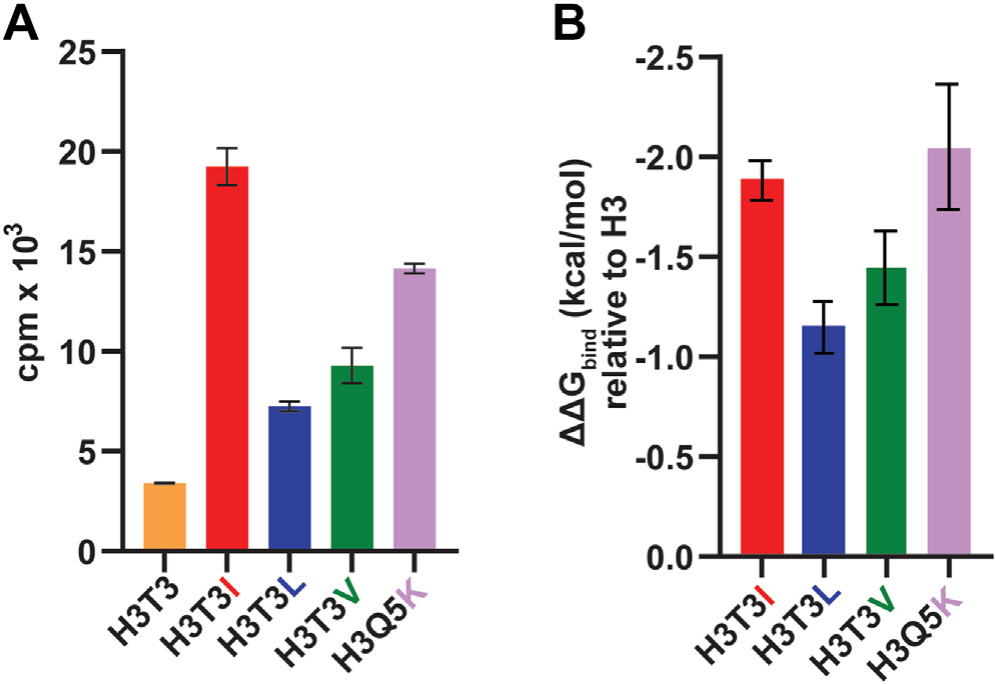
**MSλD analysis confirms PRDM9 substrate selectivity.** Comparison of *in vitro* lysine methyltransferase assays on histone H3 peptides (*A*) and changes in binding free energies (ΔΔ*G*_bind_) relative to the WT H3 peptide (T3 and Q5) calculated from MSλD simulations (*B*). Bar graphs display mean ± SD (n = 3 for *A* and n = 5 for *B*). MSλD, multi-site λ-dynamics.

Next, we evaluated how substitutions surrounding a previously reported PRDM9 substrate, H3K4, impacted PRDM9 activity. A series of H3 peptides were synthesized with the native P-1 Thr substituted with Ile, Leu, or valine (Val; V). *In vitro* methyltransferase assays using these peptides as substrates recapitulated the rank order observed in the K-OPL screen for these residues in the P-1 position (Fig. 2*A*). The Ile peptide was methylated most efficiently, followed by Val, Leu, and then Thr. Similar analysis on a peptide with the natural P+1 Glu substituted with a Lys further confirmed the preferences identified by the K-OPL substrate screen. A peptide containing Lys at the P+1 position in the context of an H3K4 peptide resulted in a significant increase in PRDM9 activity. Overall, while a Lys residue at P+1 enhances methylation of a target sequence, at the P-1 position, we observed Ile>Val>Leu>Thr to enhance peptide methylation. These results further confirm the accuracy and predictive power of the K-OPL selectivity profile for PRDM9 substrates.

### Multisite λ-dynamics analysis identifies a structural rationale for PRDM9 substrate selectivity

To determine the molecular basis for PRDM9 substrate selectivity, a computational and structural analysis of PRDM9 peptide binding was performed with multisite λ-dynamics (MSλD). MSλD is a physics-based alchemical free energy method that can provide structural insights into molecular binding *via* molecular dynamics sampling and can quantify the thermodynamic effects of a protein side chain mutation on peptide-protein binding (20, 21, 22). Using a previously reported mPrdm9 SET domain structure solved in complex with an H3K4me2 peptide and AdoHcy (19), four peptide-substrate substitutions were investigated with MSλD. It is important to note that the mPrdm9 has a high sequence identity to human PRDM9 (72.8%). Furthermore, we compared the substrate selectivity of hPRDM9 and mPrdm9 on the same H3K4 peptides used in MSλD analysis and found no differences (Fig. S2*A*).

At the P-1 position, the native Thr was mutated to Ile, Leu, and Val; then, at the P+1 position, the native glutamine (Gln; Q) was mutated to Lys. Computationally, the experimental K-OPL activity trends (Ile > Val > Leu > Thr at P-1; Lys > Gln at P+1) were reproduced (Fig. 2*B*). Additional targeted experiments confirmed these predictions and demonstrated a strong positive correlation between predicted binding affinities of different peptide mutants and their observed methylation activities by PRDM9 (Pearson’s r = 0.867; Fig. S2*B*). These results suggest a strong correlation between binding affinity for PRDM9 peptide substrates and enzymatic activity. This work also demonstrates that the MSλD analysis is effective for identifying and screening preferred peptide substrates for PRDM9 and may be beneficial for screening other KMTs.

**Figure 3:**
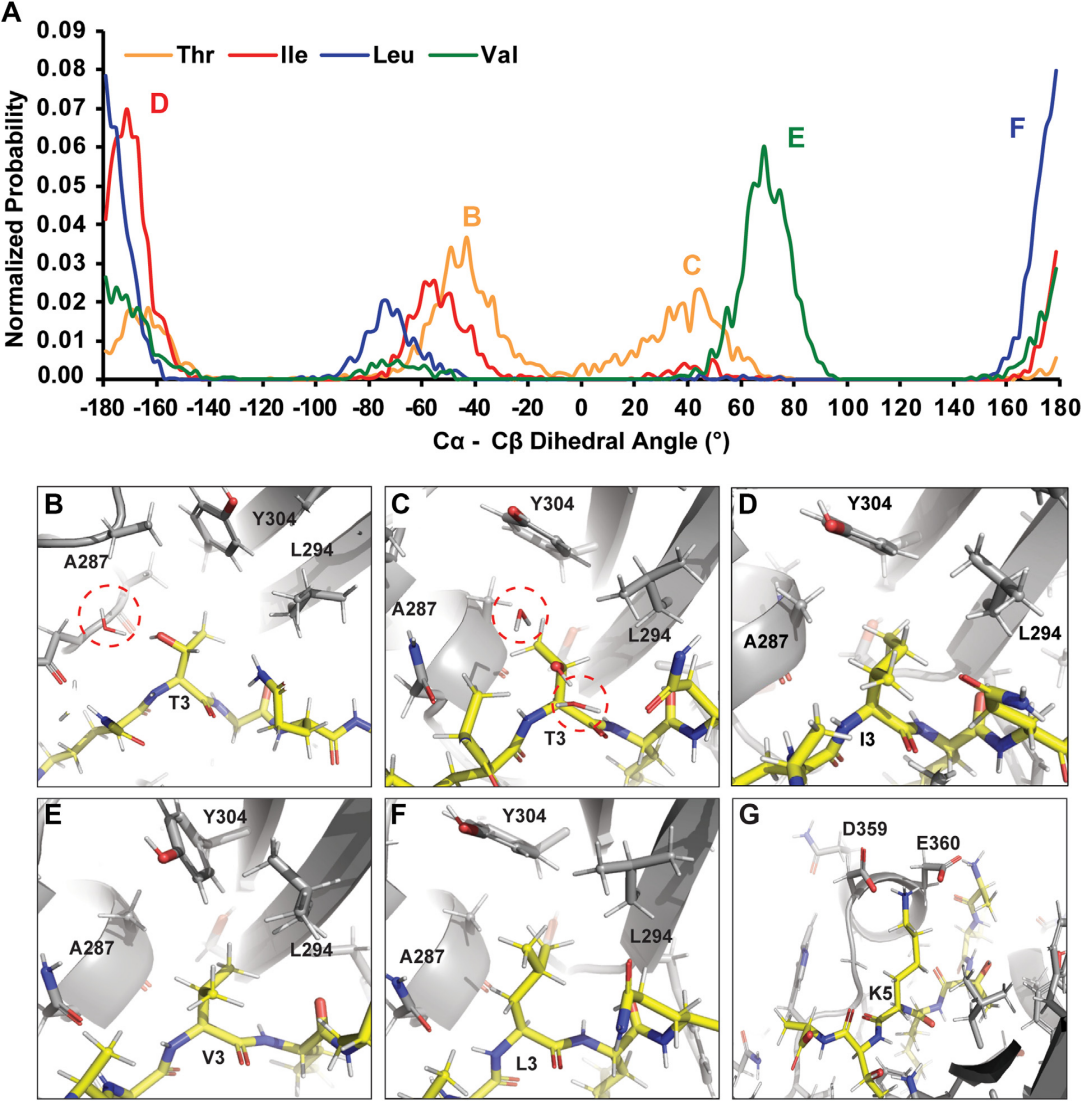
**Analysis of MSλD trajectories reveals structural rationale for PRDM9 substrate selectivity.***A*, distribution of Cα-Cβ dihedrals from MSλD. Representative images from MSλD trajectories for native Thr3 (*B* and *C*) or substitutions Ile3 (*D*), Val3 (*E*), Leu3 (*F*), or Lys5 (*G*). MSλD, multi-site λ-dynamics.

MSλD trajectories were analyzed to determine the molecular features that drive PRDM9 preference for Ile in the P-1 position and Lys in the P+1 position. A comparison of the distance between the ε-amine of the substrate lysine and SAM showed no significant changes (Fig. S3*A*), suggesting no direct influence on the catalytic proficiency of the enzyme by the different substitutions. Rather, an induced fit model of complementary size, shape, and nonbonded interactions was observed. Analysis of the Cα-Cβ dihedral angles at the P-1 position indicated that each residue had slightly different rotational preferences when bound to PRDM9. Thr adopts two primary conformations to form a hydrogen bond with the backbone carbonyl group of Prdm9 Ala287 or, when rotated ∼85°, to expose the hydroxyl group to solvent (Fig. 3, *A*–*C*). In either conformation, Thr suffers a slight desolvation penalty for binding PRDM9, since it is partially shielded by PRDM9 and can form only 1 to 2 hydrogens bonds at a time (approximately one fewer hydrogen bond than Thr would form in bulk solvent). In contrast, Val, Ile, and Leu show favorable hydrophobic packing against PRDM9 residues Ala287, Tyr304, and Leu294 at the P-1 position (Fig. 3, *A*, *D*, *E*, and *F*). Ile is slightly favored over Val due to its increased size and interactions with these hydrophobic residues and its ability to extend into the back of the P-1 pocket with its Cδ atom, though both Ile and Val branch at Cβ to bind similarly as Thr and populate similar Cα-Cβ dihedral angles (Fig. 3, *D* and *E*). Leu is the least preferred of the hydrophobic residues tested due to minor steric clashes made with Tyr304. This is evident in the longer average distances for Leu between its Cα atom and the Y304 aromatic ring center of mass compared to those for Thr, Ile, or Val (Fig. S3*C*). This is largely due to leucine’s branching at the Cγ atom, which prevents it from extending into the P-1 pocket, like Ile, and slightly alters its conformational preferences in the Cα-Cβ (Fig. 3*F*) and Cβ-Cγ (Fig. S3*B*) dihedral angle distributions. Finally, the preference for Lys in the P+1 position is readily explained by the introduction of favorable ionic interactions between the P+1 Lys with residues Glu360 and Asp359 (Fig. 3*G*). This also explains why Arg would be favorable at the P+1 position, as it will likely form similar favorable ion–ion interactions. Overall, this MSλD analysis provides a structural rationale for PRDM9 substrate selectivity that supports the K-OPL–derived substrate selectivity profile and provides mechanistic insight into the striking preference for Ile in the P-1 position and Lys at the P+1 position.

### Identification of nonhistone peptide substrates of PRDM9

**Figure 4:**
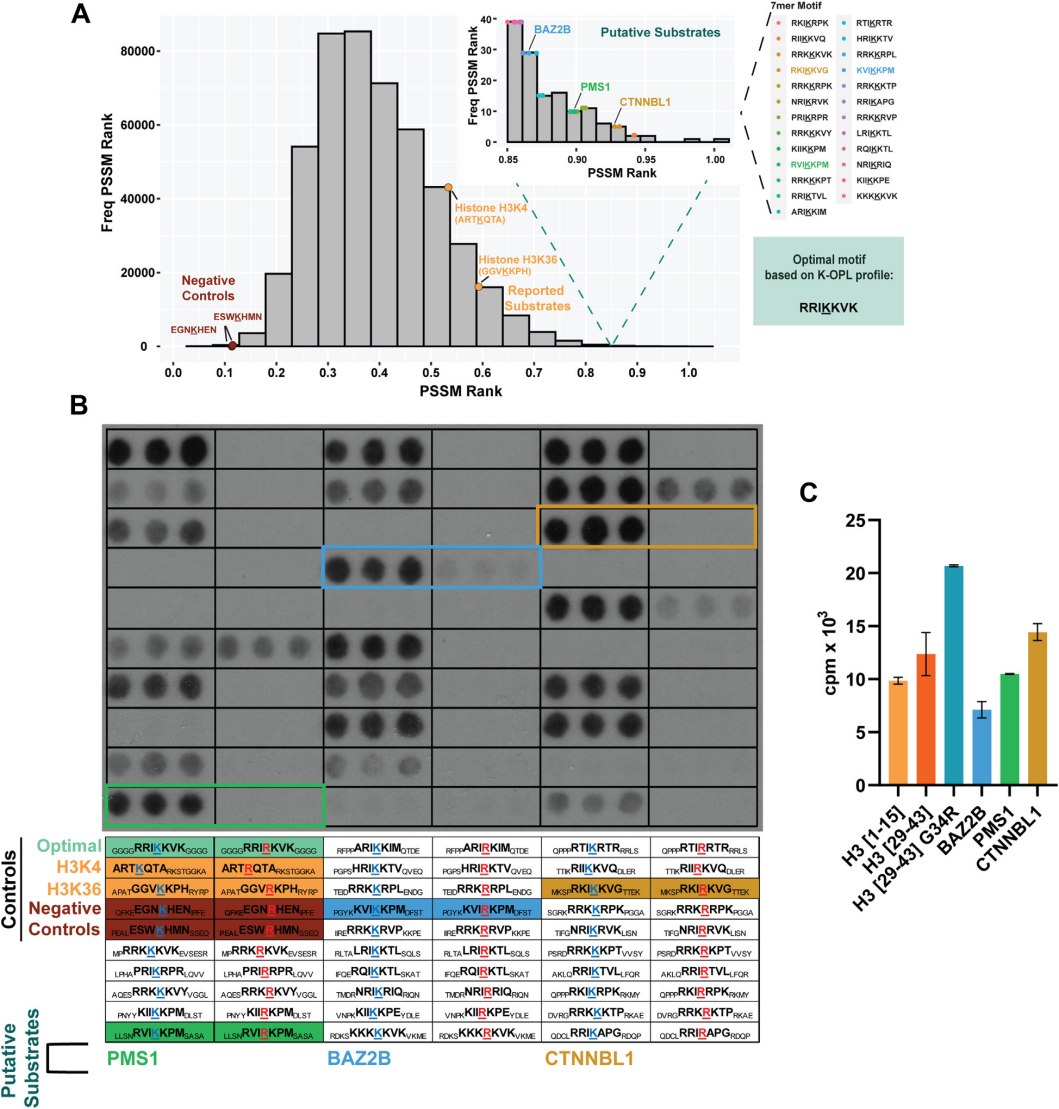
**Methylation of nonhistone protein–derived peptide substrates by PRDM9.***A*, distribution of position-specific scoring matrix (PSSM) scores for all 7-mer motifs surrounding a central lysine in the human proteome based on PRDM9 signal on K-OPL sets. Substrates used for spot array are highlighted, including negative controls with low PSSM scores (*maroon*), reported histone substrates (*orange*), and putative substrates with PSSM scores ranked in the top 15% of all scores (*inset*). *B*, fluorography signal from PRDM9 lysine methyltransferase activity on peptide spot array (15-mer peptides spotted in triplicate). One hour film exposure is shown. The location and sequence of each feature are displayed below. *C*, comparison of newly identified nonhistone substrates with histone peptides. Graph depicts mean (n = 2) ± SD signal from scintillation proximity methyltransferase assays (SPA) with biotin-labeled peptides. K-OPL, lysine-oriented peptide library.

We next identified and evaluated putative PRDM9 substrates based on the selectivity profile: we used the PRDM9 K-OPL selectivity data to score each lysine-centered 7-mer motif found in the human proteome (Fig. 4*A* and Table S1) and synthesized a peptide spot array to test 25 putative PRDM9 substrates. The putative substrates were chosen from the top 15% of all lysine-centered 7-mer motifs found in human proteins. The top 15% represent 137 sequence motifs, which were narrowed down to 25 based on manual curation (Table S2). Factors for inclusion included expression in spermatocytes, one of the few tissues where PRDM9 is expressed, connection to double-strand break formation or DNA repair, and/or the predicted methylated lysine residing within an annotated protein domain/region (Table S3). In addition to putative substrates, the peptide array also contained two negative control peptides, peptides containing the known histone substrates of PRDM9 (H3K4 and H3K36), and an artificial peptide containing the optimal residues identified from the K-OPL selectivity screening. Each peptide was synthesized *via* the spots method (see Experimental procedures for details) in triplicate, and to determine if other lysine residues in the peptides were being methylated, a corresponding peptide with the predicted target lysine substituted with an arginine was also included. PRDM9 methylated 18 of the 25 putative substrates, and 12 substrates showed more signal than either histone peptide control (Fig. 4*B*). All seven putative substrates that were not methylated did not contain an Ile in the P-1 position but had nearly optimal sequences in all other positions, further highlighting the importance of the P-1 Ile for PRDM9 substrate selectivity. PRDM9 methylated the H3K36 peptide more efficiently than H3K4, likely due to the P-1 Val in H3K36, which the K-OPL selectivity profile shows is preferred over the Thr found at this position in the context of H3K4. Substitution of the Lys predicted to be methylated with Arg resulted in a complete loss of any detectable methylation signal for nearly all peptides. A notable exception is ACE K722, which showed a significant signal even in the Lys-to-Arg control but also contained an additional Ile-Lys motif.

We selected three substrates for further study—BAZ2B, PMS1, and CTNNBL1—that PRDM9 robustly methylated and whose predicted methylation site is located within a functional protein domain/region. The lysine residues predicted to be methylated within BAZ2B, PMS1, and CTNNBL1 reside within the bromodomain, high mobility group box, and armadillo (ARM) repeat, respectively (Fig. S4*A*). Lysine methylation in these domains on other proteins has previously been shown to alter protein function, localization, or stability (23, 24, 25). To further test whether PRDM9 methylated these proteins, we synthesized peptides corresponding to BAZ2B, PMS1, and CTNNBL1 sequences for KMT assays in solution. PRDM9 methylated all three peptides to a similar degree as histone peptides, with CTNNBL1 showing the highest signal (Fig. 4*C*); CTNNBL1 was also ranked the highest of these three putative substrates (Fig. 4*A*, inset) according to the K-OPL selectivity profile. All three substrates contained the P-1 Ile and P+1 Lys validated as critical residues for PRDM9 substrate activity.

In addition to testing these putative substrates, we also synthesized a peptide centered around H3K36 with Gly 34 substituted with Arg, a variant found in glioblastoma and osteosarcoma (26, 27, 28, 29). Structural studies of SETD2, another KMT that mediates H3K36 trimethylation (H3K36me3), showed that the bulky Arg substitution prevents substrate engagement (30). As a result, H3G34R was shown to decrease global trimethylated H3K36 on molecules with the substitution (27). However, the PRDM9 K-OPL selectivity profile predicts Arg would be preferred over Gly at this position. Indeed, PRDM9 methylated the H3G34R peptide more efficiently than WT H3K36 (Fig. 4*C*). A recent study demonstrated that PRDM9 is expressed in some glioblastomas, and studies of H3G34R have identified a redistribution of H3K36 methylation with some loci modified with a higher frequency (31). Our data underscore the possibility that other KMTs may have increased activity toward some variant histone proteins, perhaps explaining why some of these histone mutations result in a redistribution of the modification rather than decreases or increases alone (31).

### PRDM9 methylates nonhistone proteins

To determine whether PRDM9 methylates BAZ2B, PMS1, and CTNNBL1 at the protein level, we cloned and purified each protein. *In vitro* KMT assays were performed using tritiated SAM with recombinant BAZ2B, PMS1, and CTNNBL1 as putative substrates and histone H3 as a positive control. The reactions were separated on SDS-PAGE gels, treated with an enhancer, dried, and exposed to film to detect methylated proteins by fluorography. As expected, PRDM9 methylated histone H3; PRDM9 also methylated PMS1 and CTNNBL1 (Fig. S4*B*). Only a faint band was detected for BAZ2B. Analysis of available structural data for BAZ2B shows the predicted methylation motif is in an alpha-helix (PDB: 3G0L), which may explain why BAZ2B is not methylated more efficiently by PRDM9.

**Figure 5:**
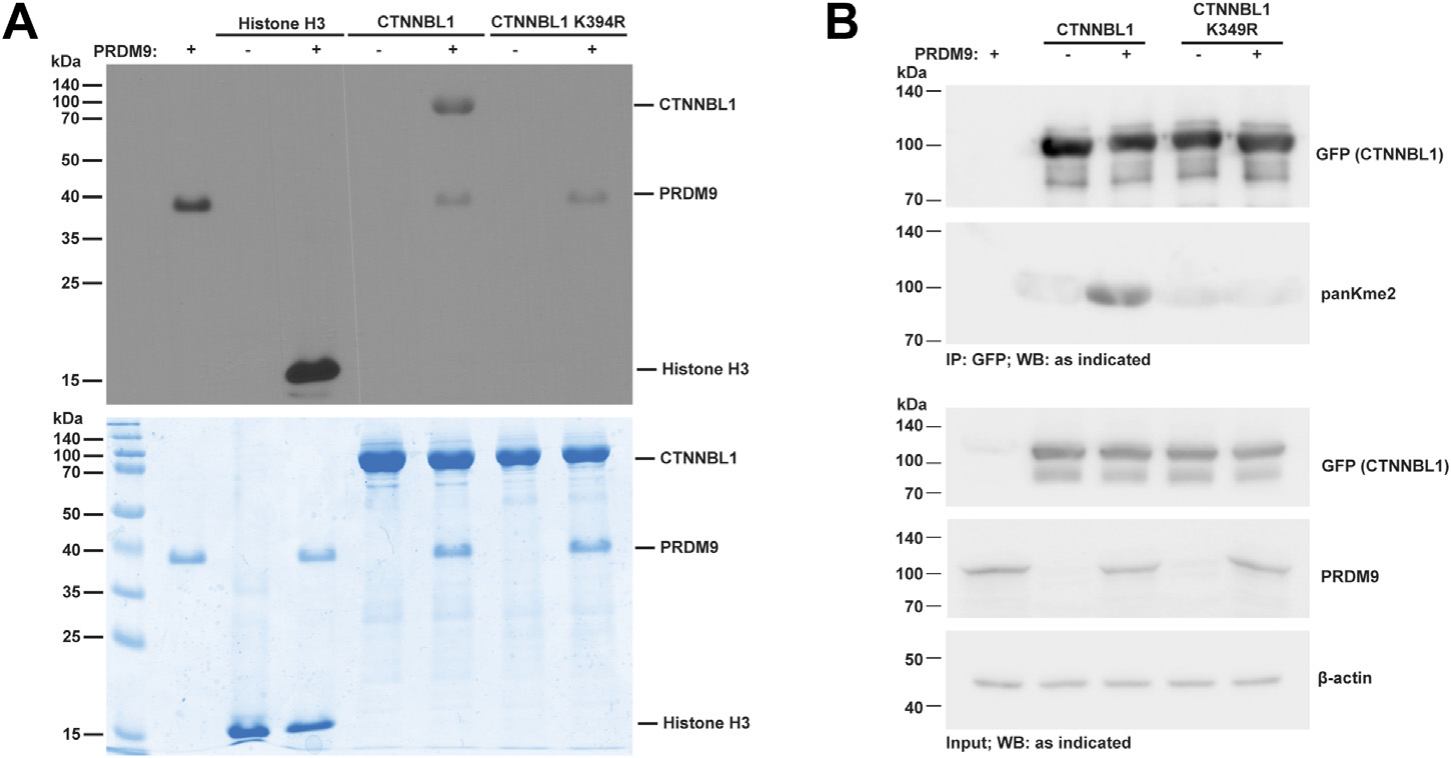
**PRDM9 methylates nonhistone proteins.***A*, comparison of PRDM9 methylation of CTNNBL1 when the target Lys is substituted with an Arg (K394R). Representative of three independent experiments. *Top panel* depicts fluorography signal after *in vitro* methyltransferase reactions (see Experimental procedures), and the *bottom panel* shows total protein stained with Coomassie after SDS-PAGE. *B*, methylation of CTNNBL1 by PRDM9 coexpressed in HEK293T cells detected by immunoblot as indicated. Representative of three independent experiments.

Since PRDM9 displayed marked KMT activity on CTNNBL1, we sought to validate that the fluorography signal on CTNNBL1 was due to methylation of the lysine predicted to be methylated, K394. We performed *in vitro* KMT assays using recombinant CTNNBL1 with and without the target lysine substituted with arginine (K394R) (Fig. 5*A*). PRDM9 methyltransferase activity on WT CTNNBL1 is evident; in contrast, there was no detectable activity on CTNNBL1 K394R, supporting the conclusion that PRDM9 methylates CTNNBL1 at K394. When comparing the activity of PRDM9 on CTNNBL1 to that on histone H3, it is important to note that PRDM9 methylates CTNNBL1 at one residue and methylates histone H3 at multiple residues, which could contribute to the higher fluorography signal on histone H3.

Strong auto methylation signal was observed on PRDM9 in the absence of any substrate (Figs. S4*B* and 5*A*). However, in the presence of histone H3, no auto methylation was detected, whereas, in the presence of nonhistone substrates that PRDM9 methylated, there was a reduction in auto methylation. Addition of BAZ2B, which PRDM9 did not methylate, had no impact on auto methylation. Interestingly, despite detecting no methylation on CTNNBL1 K394R, PRDM9’s auto methylation was reduced compared to PRDM9 alone (Fig. 5*A*), suggesting that PRDM9 may still bind to CTNNBL1, preventing auto methylation. There are three lysine residues preceded by isoleucine in full-length PRDM9, suggesting that auto methylation could impact PRDM9 function.

Next, we evaluated whether PRDM9 methylates nonhistone proteins in cells. PRDM9 is usually expressed in germ cells under specific developmental contexts, but a recent study identified overexpression of PRDM9 across human cancers (13, 16). Thus, we chose overexpression of PRDM9 in HEK293T cells as a model of PRDM9 overexpression that may occur in different disease contexts. GFP-tagged CTNNBL1 was expressed with or without full-length PRDM9 in HEK293T cells (Fig. 5*B*). CTNNBL1 expression levels were not impacted by PRDM9 expression. CTNNBL1 was immunoprecipitated and analyzed by Western blot using a pan dimethyl-lysine antibody. CTNNBL1 reacted with this antibody in all samples, but in cells also expressing PRDM9, there was a significant increase in signal, indicating PRDM9 mediates CTNNBL1 methylation within cells. However, no increase in methylation signal was detected when CTNNBL1 was expressed with the target lysine mutated to arginine (CTNNBL1 K394R). Overall, these results suggest PRDM9 mediates the methylation of CTNNBL1 at K394 both *in vitro* and in cells.

## Conclusions

The data presented in this study suggest that PRDM9 may have physiologically relevant nonhistone substrates. *In vitro* K-OPL screening revealed that PRDM9 prefers to methylate a sequence motif not found in any histone protein, and a structural rationale for PRDM9 selectivity was provided by MSλD computational analysis. Furthermore, we have demonstrated that PRDM9 methylates CTNNBL1 at K394 both *in vitro* and in cells. Lysine 394 resides within an ARM repeat region of CTNNBL1; ARM repeats form an α-superhelix, providing a platform for protein interactions, including activation or degradation of the target protein (32). In the case of the related protein β-catenin, regulation of lysine methylation within an ARM repeat region was shown to regulate protein stability (24). Future studies are necessary to determine the functional impacts of CTNNBL1 methylation.

In previous studies aimed at determining the sequence selectivity of other KMTs, a sequence component from previously identified substrates has typically been confirmed as critical for substrate selectivity (6). PRDM9 surprisingly preferred a motif not found in any histone protein, raising the intriguing question: why does PRDM9 prefer this motif? One potential answer is that PRDM9 methylates nonhistone substrates that have not been identified. Another possibility is that the cellular context may influence which substrates PRDM9 methylates, and many KMTs are part of multicomponent protein complexes. While the inherent sequence determinants of substrate selectivity identified from our experiments remain the same, complex affiliation or additional factors can influence which substrates are methylated. Efforts have been made to determine interacting partners of PRDM9 within mouse spermatocytes, revealing that the PRDM9 KRAB domain interacts with CXXC1, EWSR1, EHMT2, and CDYL (33). Less is known about PRDM9-interacting partners in cancer. Future studies to evaluate how PRDM9 interactions impact the methylation of histone and nonhistone substrates will be critical to answering this question definitively. However, the PRDM9 K-OPL selectivity profile derived herein will be a helpful resource to identify whether PRDM9 has different nonhistone substrates in disease and developmental contexts.

## Experimental procedures

### Protein expression and purification

Recombinant human PRDM9 (amino acids 191–415) with an N-terminal GST tag (Active Motif) was used for experiments shown in Figure 1 and Fig. S1; all other experiments were performed using recombinant PRDM9 purified as follows. Human PRDM9 (amino acids 195–385), mouse Prdm9 (amino acids 198–368), BAZ2B (amino acids 2062–2166), PMS1 (amino acids 333–705), and CTNNBL1 (full length, amino acids 1–563) were cloned in a pET28b expression vector as 6xHis-SUMO N-terminal fusions. Point mutations were generated by QuikChange site-directed mutagenesis (Stratagene). Constructs were transformed into BL21(DE3) cells and plated on LB agar plates containing kanamycin. A single colony was selected to grow a starter culture in LB media with kanamycin overnight at 37 °C. The starter culture was diluted 100-fold into 1 l of LB media in a 2 l baffled shaker flask and grown at 37 °C shaking at 160 rpm until the *A*600 (absorbance at 600 nm) reached 0.6 to 0.8, at which point the temperature was lowered to 16 °C, IPTG was added (0.5 mM), and incubation was continued overnight with shaking at 160 rpm. Bacteria were harvested by centrifugation and either frozen at −80 °C or used immediately. Cells were resuspended in lysis buffer (20 mM Tris pH 8.0, 500 mM NaCl, 5 mM imidazole, 1 mM DTT, 1 mM PMSF) and lysed by passing through a microfluidizer. Cell lysates were cleared by centrifugation at 14,000*g* for 30 min. After incubating cleared lysate with Pierce Ni-IMAC resin for 1 h at 4 °C, the resin was washed with lysis buffer and bound protein was eluted using elution buffer (20 mM Tris pH 8.0, 500 mM NaCl, 500 mM imidazole, 1 mM DTT, 1 mM PMSF). Eluted protein was concentrated and buffer exchanged using a 10K MWCO Amicon ultra centrifugation device, followed by size-exclusion chromatography (SEC) on a Cytiva Superdex 200 Increase column using an AKTA Pure FPLC and SEC buffer (50 mM Tris pH 8.0, 150 mM NaCl). Fractions were separated by SDS-PAGE and stained with Coomassie. Fractions containing the predicted proteins were concentrated using an Amicon Ultra Centrifugation device, aliquoted, and stored at −80 °C in SEC buffer.

### Lysine methyltransferase surface proximity assay

The K-OPL sets were synthesized by Pepscan as C-terminal PEG-biotin conjugates. All other peptides were purchased from Biomatik. Except when otherwise stated, for reactions with K-OPL sets or biotin-labeled peptides, reactions (10 μl) containing 0.8 μg of PRDM9, 0.5 μg of a substrate, and 2.2 μCi of ^3^H-SAM (PerkinElmer) in KMT reaction buffer [50 mM tris (pH 8.8), 5 mM MgCl_2_, and 4 mM DTT] were incubated for 6 h at room temperature. Reactions were stopped by adding TFA to a final concentration of 0.5%, neutralized by diluting with 135 μl of 50 mM NaHCO3, and transferred to white 96-well microplates (PerkinElmer). Eight microliters of streptavidin-coated SPA bead slurry (0.1 mg/μl, PerkinElmer) were added to each well, the plate was sealed using PerkinElmer TopSeal-A, and centrifuged for 3 min at 1000 rpm. The bead slurry was incubated with the reaction mixture for 30 min before liquid scintillation counting for 1 min using a Hidex Sense Beta microplate reader. K-OPL peptides with high, medium, and low signal were selected for positions P-3, P-1, and P+1. In each case, the fixed amino acid K-OPL set with the highest signal was selected as the high signal group. The fixed amino acid K-OPL set with approximately half the signal was selected as the medium example and the low signal was a K-OPL set with signal near the background.

### Peptide spot assays

Putative PRDM9 substrates were synthesized as 15-mer peptides on a β-alanine derivatized membrane (Amino-PEG500-UC540 membrane, CEM Corporation) using N-(9-fluorenyl) methoxycarbonyl (Fmoc)-protected activated amino acids (CEM Corporation) and a solid phase synthesis protocol as described (34, 35). The membranes were deprotected with a deprotection cocktail that includes 88% TFA, 5% water, 5% phenol, and 2% triisopropylsilane, followed by three washes with dichloromethane, three washes with dimethyl formamide, and three washes with ethanol as described (34, 35). The deprotection protocol was repeated twice, and the membrane was allowed to dry and stored at −20 °C. Putative PRDM9 substrates (15-mer peptides) were synthesized in triplicate, and peptides with the lysine residue predicted to be methylated substituted with arginine were included as a control. To test PRDM9 methylation activity on putative substrates, the peptide array was first preincubated in KMT reaction buffer [50 mM tris (pH 8.8), 5 mM MgCl_2_, and 4 mM DTT, 0.01% Triton X-100] for 20 min; following which, the membrane was incubated in KMT reaction buffer supplemented with ^3^H-SAM (PerkinElmer) and 6xHis-SUMO–tagged human PRDM9 (0.5 μCi and 0.4 μg, respectively, per 10 μl reaction) for 1 h at room temperature. The membrane was then washed five times for 5 min in washing buffer (1% SDS in PBS) and incubated in Amplify fluorographic reagent (Cytiva) for 5 min. All incubation and washing steps were carried out using a shaker. The membrane was exposed to film at −80 °C in the dark and the film was exposed following a range of exposure times as indicated.

### *In vitro* lysine methyltransferase reactions

For reactions with protein substrates, 0.125 μg of PRDM9, 1 μg of the indicated substrates, and 1 μCi of ^3^H-SAM (PerkinElmer) in KMT reaction buffer were incubated for 1 h at room temperature. Purified human histone H3 protein was purchased from Active Motif. Reactions were quenched by the addition of SDS loading buffer and resolved by SDS–PAGE. Following the detection of total protein by Coomassie staining, gels were treated with EN3HANCE (PerkinElmer), dried, and methylated proteins were detected by fluorography.

### MSλD analysis

A structural rationale for PRDM9’s observed K-OPL–binding preferences was investigated computationally with MSλD (20, 21, 22). A previously reported mouse Prdm9 SET domain structure (PDBID: 4C1Q) with an H3K4me2 peptide and AdoHcy was used to provide a structural model of holo human PRDM9 (19). To reverse the catalytic reaction, AdoHcy was converted to SAM, and the H3K4me2 peptide was reverted to its mono-methylated form (H3K4me1). Potential residue flips and protonation state assignments for titratable groups were determined with the assistance of Molprobity and ProPKa (36, 37, 38). The system was then solvated with the CHARMM-GUI webserver (39); all crystallographic solvent molecules were retained and a cubic box of TIP3P water molecules was generated to solvate the peptide–PRDM9 complex (39). A neutralizing buffer of 0.1 M of Na^+^Cl^−^ was also added to the simulation cell (40). A similar setup was followed for preparing a solution of the isolated peptide. All CHARMM-based force fields were used to represent the components of the chemical system, including CHARMM36 for protein and peptide molecules and CGenFF for the SAM cofactor (41, 42, 43, 44, 45). MSλD free energy calculations were run in the CHARMM molecular simulation package on graphical processor units with BLaDE (45, 46, 47). All simulations were run in the isothermal-isobaric ensemble at 25 °C and 1 atm. SHAKE was used to restrain all heavy-atom-hydrogen bond lengths (46). Long-range interactions were gradually smoothed to zero with force switching from 9 to 10 Å, and particle mesh Ewald was used to correct for long-range electrostatic interactions (48, 49, 50, 51). Soft-core nonbonded potentials were used to avoid endpoint singularities for alchemical sampling (52). Prior to MSλD production sampling, each system was subject to 250 steps of steepest descent minimization to remove potential clashes. Biasing potentials for MSλD were then determined with the adaptive landscape flattening algorithm over a combined 173 ns of sampling (52). To determine the final relative free energy differences, five replicate 25 ns MSλD production simulations were performed. In accordance with a standard alchemical thermodynamic cycle for calculating binding affinities, residue mutations were investigated in both isolated peptide and peptide PRDM9–bound states (19). Additionally, for the Gln to Lys mutation at the P+1 position of the peptide substrate, the alchemical ion approach was used to maintain charge neutrality throughout the duration of a charge-changing MSλD perturbation. The alchemical ion–water pair was restrained with a harmonic force constant of 59.2 kcal/mol⋅Å^2^ (53, 54). All dihedral and distance analyses were performed with CHARMM, and trajectory analyses and figures were made with PyMOL (https://pymol.org/2/support.html?).

### Bioinformatics analysis

A position-specific scoring matrix (PSSM) score for PRDM9 selectivity was calculated for every lysine-centered 7-mer in the human proteome based on PRDM9 activity on K-OPL sets. The input for PSSM score calculation was a normalized and transformed matrix of PRDM9 signal on K-OPL positions P-3 to P+3. The average cpm of three independent measurements was globally normalized to the highest cpm value, a pseudocount (+1) was added to each normalized count, and then the natural log was taken. PSSM scores were calculated on the normalized and transformed matrix by summing the score for each amino acid at each position ±3 residues from a central lysine.

### Cell culture

HEK293T cells (ATCC CRL-3216) were cultured in Dulbecco’s modified Eagle’s medium (Corning) supplemented with 10% fetal bovine serum (Sigma) and antibiotic/antimycotic (Corning) in an incubator at 37 °C and 5% CO_2_. Analysis of CTNNBL1 methylation was performed by transfecting N-terminally GFP-tagged full-length human CTNNBL1 or N-terminally FLAG-tagged full-length human PRDM9 cloned into pcDNA3.1 vectors (Genscript) using X-tremeGENE 360 (Roche). After 48 h, cells were harvested by trypsinization, washed in PBS, and resuspended in lysis buffer (10 mM Pipes [pH7.0], 300 mM sucrose, 100 mM NaCl, 3 mM MgCl_2_, 0.1% Triton X-100, 1x Universal Nuclease, and 1x protease inhibitor cocktail). Total protein was quantified by Bradford assay (Bio-Rad). Immunoprecipitation of GFP-tagged CTNNBL1 was performed using a magnetic GFP-Trap (Bulldog Bio) per the manufacturer’s instructions, and samples were eluted by boiling in SDS-PAGE sample buffer (Bio-Rad). Inputs and immunoprecipitated samples were resolved by SDS-PAGE, transferred to polyvinylidene difluoride membrane (Thermo Fisher Scientific), and probed with the indicated primary antibodies (PRDM9 [Sigma; catalog no.: ABE1947], GFP [ProteinTech; catalog no.: 50430-2-AP], β-actin [Cell Signaling Technologies; catalog no.: 3700S], and panKme2 [PTM Biolabs; catalog no.: PTM-606]). Membranes were washed in PBS-tween and probed with horseradish peroxidase-conjugated secondaries (Cytiva) prior to imaging using a Bio-Rad Chemidoc.

## Data availability

All data are included in the manuscript and supporting information.

## Supporting information

This article contains supporting information.

## Conflict of interest

The authors declare that they have no conflicts of interest with the contents of this article.
