# Supplementary material for: Identification of nonhistone substrates of the lysine methyltransferase PRDM9
Source: J Biol Chem. 2023 Mar 25;299(5):104651. doi: 10.1016/j.jbc.2023.104651 (PMC10164904; doi:10.1016/j.jbc.2023.104651)
Supplement: Supporting Figures S1–S4 [file mmc2.pdf]

Supplementary Materials for

Identification of nonhistone substrates of the lysine methyltransferase PRDM9

Jocelyne N. Hanquier[1,2], Kenidi Sanders[1,3], Christine A. Berryhill[1], Firoj K. Sahoo[5], Andy Hudmon[5], Jonah Z. Vilseck[1,3,4], Evan M. Cornett[1-4,*]

[1]Department of Biochemistry and Molecular Biology, [2]Stark Neuroscience Research Institute, [3]Melvin and Bren Simon Comprehensive Cancer Center, [4]Center for Computational Biology and Bioinformatics, Indiana University School of Medicine, Indianapolis, IN 46202, U.S.A.; [5]Department of Medicinal Chemistry and Molecular Pharmacology, College of Pharmacy, Purdue University, West Lafayette, IN 47907 U.S.A.

The PDF file includes:

Supplementary Fig. 1: Analysis of PRDM9 substrate selectivity using a K-OPL

Supplementary Fig. 2: Evaluation of the "pan"-ness of pan-specific anti-lysine methyl antibodies.

Supplementary Fig. 3 Additional MSλD trajectory analyses of PRDM9 substrate selectivity.

Supplementary Fig. 4: Domain maps of putative PRDM9 non-histone substrates and *in vitro* methyltransferase assays using recombinant proteins.

Other Supplementary Material includes the following:

Supplementary Table 1: Score of all 7-mer motifs in human proteins based on PRDM9 K-OPL data.

Supplementary Table 2: Layout of peptide spot array (Figure 4B)

Supplementary Table 3: Characterization of peptide spot array substrates.

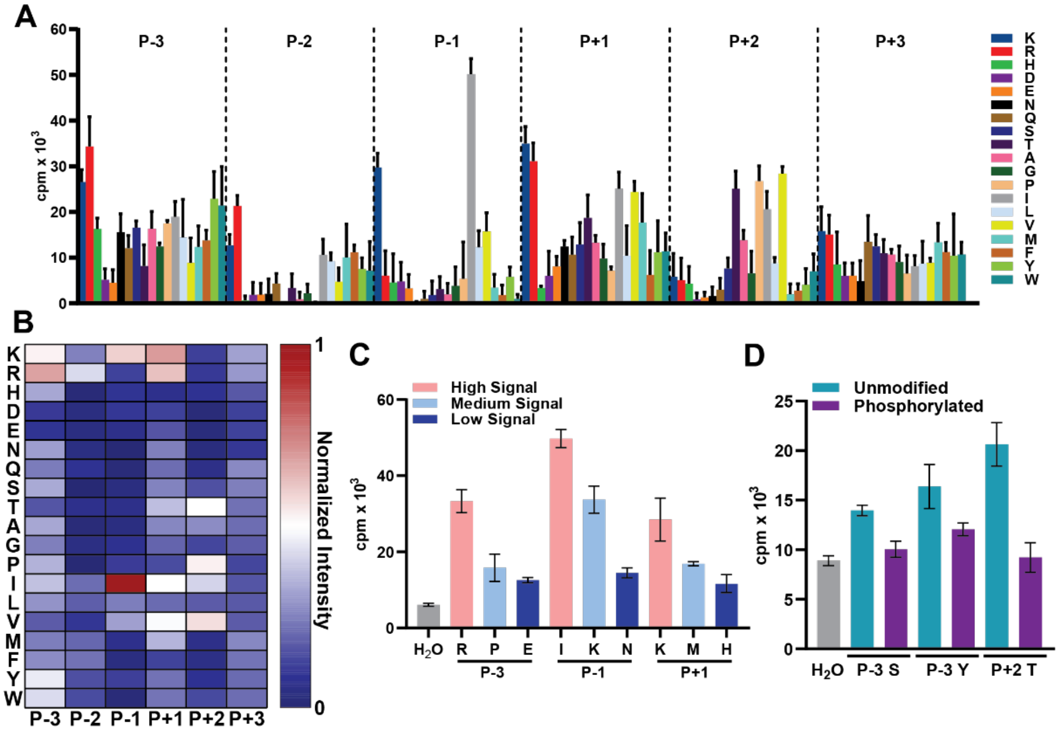

Figure S1. Analysis of PRDM9 substrate selectivity using a K-OPL. *A,* Data for PRDM9 K-OPL selectivity profile showing average signal for reactions with each K-OPL set (n=3). *B,* Heatmap normalized globally to the set with the highest signal. *C,* Validation of PRDM9 K-OPL selectivity profile on select K-OPL sets (n=2). *D,* PRDM9 reactions on phosphorylated K-OPL sets (n=3). Bar graphs display mean ±SD.

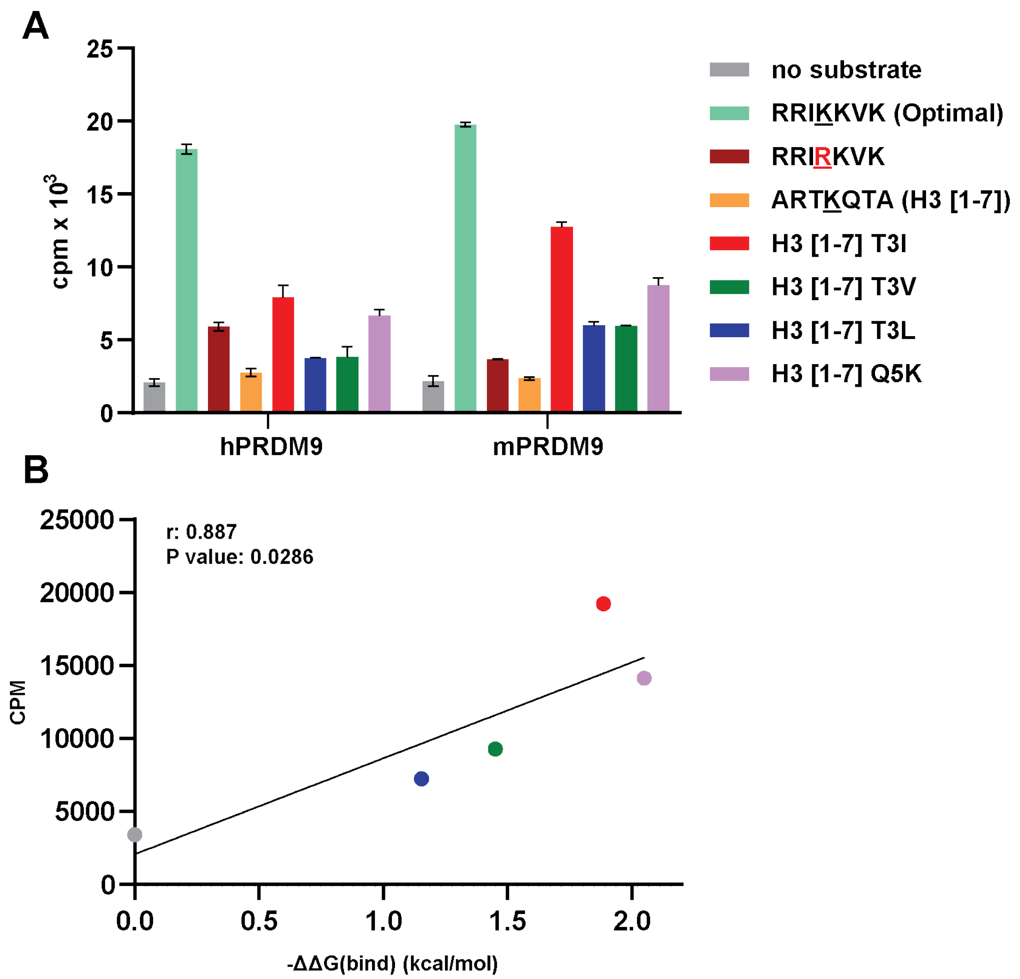

Figure S2. Analysis of PRDM9 substrate selectivity using a K-OPL *A,* Comparison of human PRDM9 and mouse Prdm9 on peptide substrates shows the highly homologous PR/SET domain have the same substrate selectivity on peptides used in this study. Graph depicts mean (n=2) ±SD signal from scintillation proximity methyltransferase assays (SPA) with biotin labeled peptides. *B,* Correlation of changes in relative binding free energies determined from MSλD for H3 peptides substituted at critical positions with experimental changes in methyltransferase activity for PRDM9. On-tailed Pearson (r) correlation was calculated using GraphPad Prism 9.

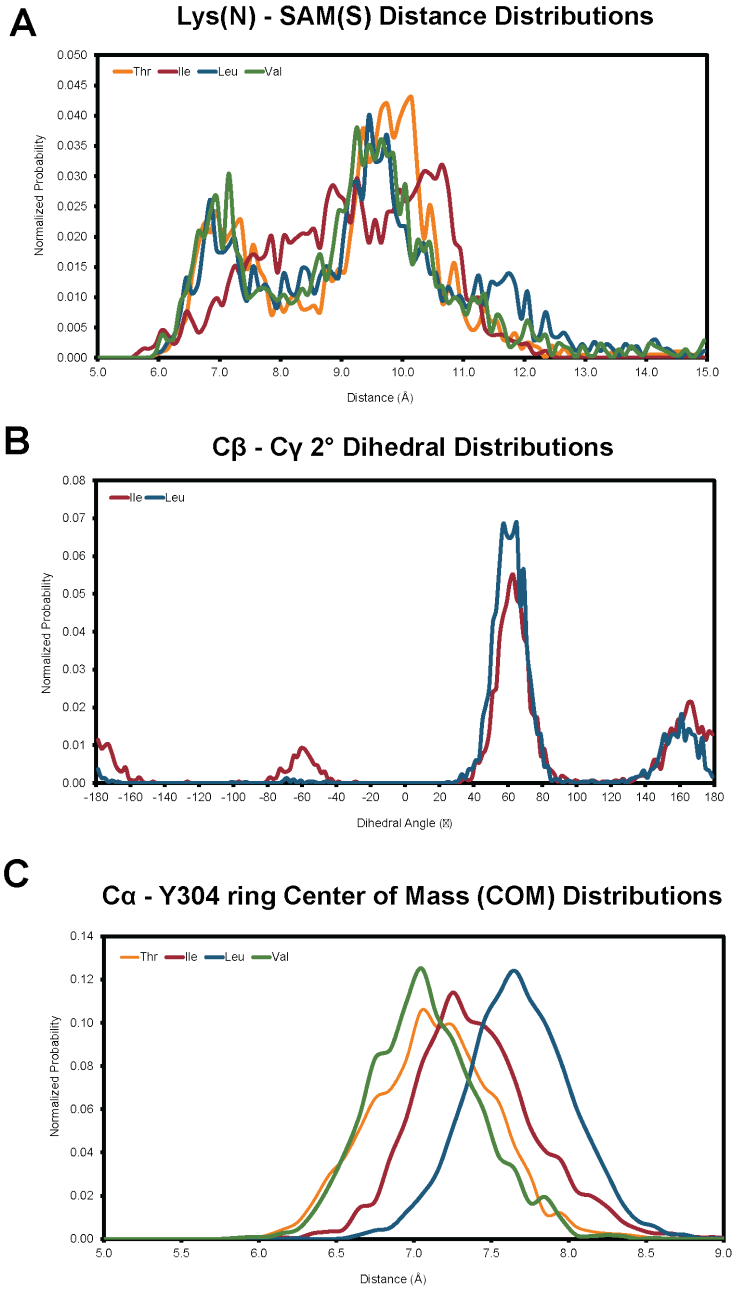

Figure S3. Additional MSλD trajectory analyses of PRDM9 substrate selectivity. *A,* Lys(N) - SAM(S) Distance Distributions. *B,* Cβ - Cγ 2° Dihedral Angle Distributions. *C,* Cα - Y304 ring Center of Mass (COM) Distance Distributions.

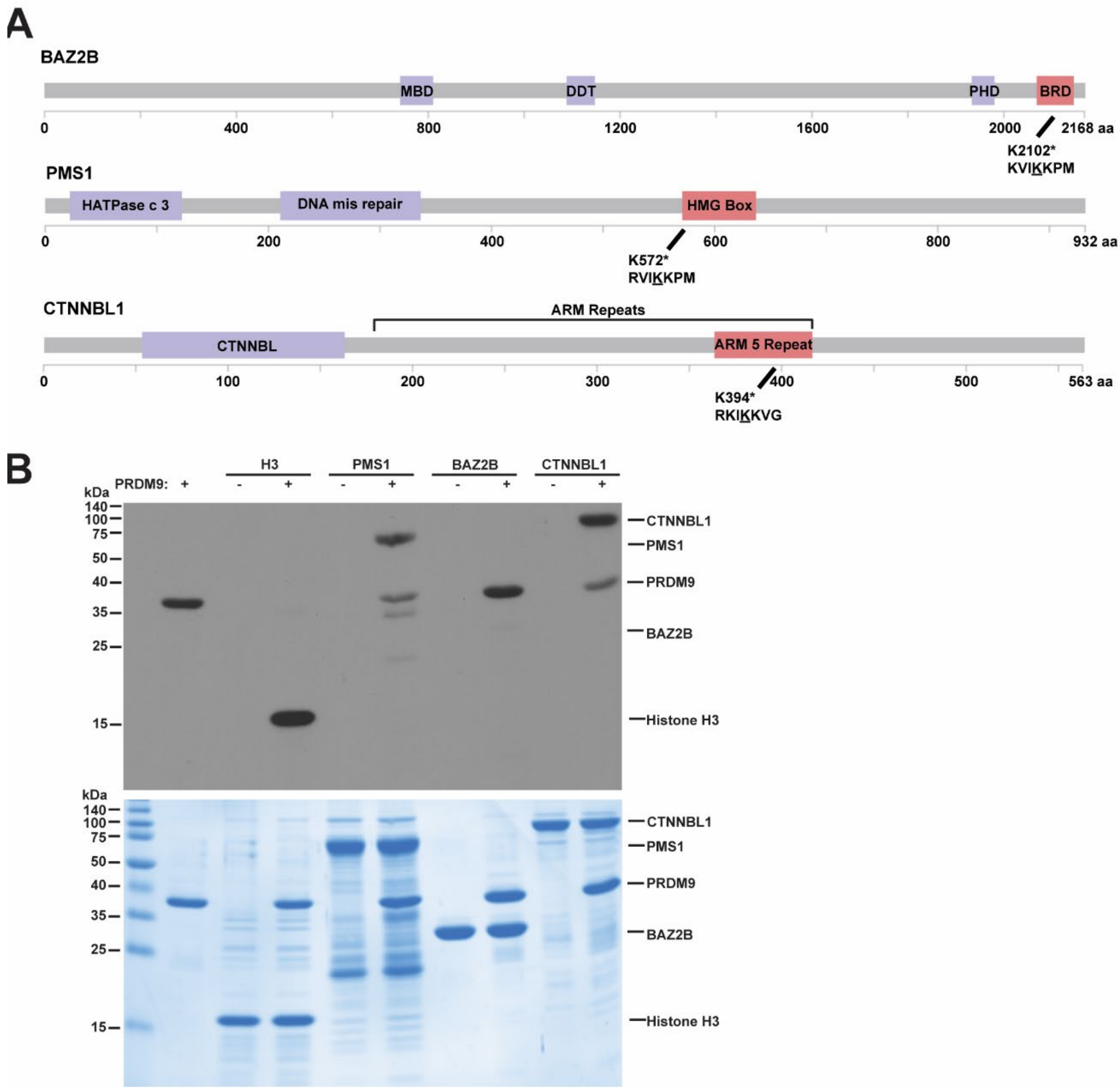

Figure S4. Domain maps of putative PRDM9 non-histone substrates and *in vitro* methyltransferase assays using recombinant proteins. *A*, The predicated PRDM9 methylation site and 7-mer motif is displayed. Data for domain boundaries was derived from UniProt. *B,* PRDM9 activity on recombinant non-histone substrates detected by fluorography (top panel). Bottom panel shows total protein stained with Coomassie after separation by SDS-PAGE. Representative of two independent experiments.
